# Accurate Segmentation of Vegetation in UAV Desert Imagery Using HSV-GLCM Features and SVM Classification

**DOI:** 10.3390/jimaging12010009

**Published:** 2025-12-25

**Authors:** Thani Jintasuttisak, Patompong Chabplan, Sasitorn Issaro, Orawan Saeung, Thamasan Suwanroj

**Affiliations:** Department of Computer Innovation and Digital Industry, Faculty of Industrial Technology, Nakhon Si Thammarat Rajabhat University, Nakhon Si Thammarat 80280, Thailand; patompong_cha@nstru.ac.th (P.C.); sasitorn_iss@nstru.ac.th (S.I.);

**Keywords:** vegetation segmentation, HSV color space, GLCM texture features, support vector machine, drone imagery

## Abstract

Segmentation of vegetation from images is an important task in precision agriculture applications, particularly in challenging desert environments where sparse vegetation, varying soil colors, and strong shadows pose significant difficulties. In this paper, we present a machine learning approach to robust green-vegetation segmentation in drone imagery captured over desert farmlands. The proposed method combines HSV color-space representation with Gray-Level Co-occurrence Matrix (GLCM) texture features and employs Support Vector Machine (SVM) as the learning algorithm. To enhance robustness, we incorporate comprehensive preprocessing, including Gaussian filtering, illumination normalization, and bilateral filtering, followed by morphological post-processing to improve segmentation quality. The method is evaluated against both traditional spectral index methods (ExG and CIVE) and a modern deep learning baseline using comprehensive metrics including accuracy, precision, recall, F1-score, and Intersection over Union (IoU). Experimental results on 120 high-resolution drone images from UAE desert farmlands demonstrate that the proposed method achieves superior performance with an accuracy of 0.91, F1-score of 0.88, and IoU of 0.82, showing significant improvement over baseline methods in handling challenging desert conditions, including shadows, varying soil colors, and sparse vegetation patterns. The method provides practical computational performance with a processing time of 25 s per image and a training time of 28 min, making it suitable for agricultural applications where accuracy is prioritized over processing speed.

## 1. Introduction

In agricultural remote sensing, accurate segmentation of green vegetation from UAV imagery is essential for precision agriculture applications in arid and desert environments. Unlike vegetated regions with dense canopy cover, desert agriculture presents unique challenges including sparse vegetation patterns, high soil-vegetation contrast, and extreme illumination variations that complicate automated segmentation. The information of the green vegetation areas can be used to estimate the percentage of the land covered by the plants [1,2], for identifying crops and weeds [3], and for analyzing the stage of crop growth in a crop growth monitoring system [4].

In remote sensing, satellite and UAV platforms provide complementary capabilities for vegetation monitoring. Satellite systems such as Sentinel-2 provide systematic wide-area monitoring with 5-day revisit frequency but offer insufficient spatial resolution for individual plant discrimination in sparse desert vegetation [5]. UAV platforms deliver ultra-high spatial resolution essential for plant-level detection in precision agriculture, though cough constrained by limited flight endurance and coverage area [6,7]. For small-scale desert agriculture where plant-level discrimination is critical and operational constraints are manageable, UAV imagery provides the necessary spatial detail for accurate vegetation segmentation. The images captured by UAVs have been widely used in many applications of precision agriculture such as crop health monitoring [8,9], agricultural surveillance [10], and yield estimation [11,12].

In recent studies, there have been several green vegetation segmentation methods proposed by researchers. The typical methods are implemented based on thresholding and using the visible spectral index. Otsu’s thresholding method [13] is widely used for separating green vegetation from the background by converting the original RGB input image to a grayscale image and using the gray level to calculate the optimal threshold value. The visible spectral index-based methods, such as ExG (Excess Green Index) [14], ExR (Excess Red Index) [15], ExGR (Excess Green minus Excess Red Index) [16], and CIVE (Color Index of Vegetation Extraction) [17], are another approach that is widely used for segmenting the green vegetation areas. They are processed based on RGB color space. However, RGB color space exhibits significant limitations for vegetation segmentation under varying illumination due to coupled chrominance and luminance information [18,19]. HSV color space was selected because it explicitly separates hue from brightness, enabling independent illumination normalization essential for robust segmentation in desert environments with extreme lighting variations [20].

Recent advances in deep learning have substantially improved vegetation segmentation accuracy. Methods such as U-Net [21] and SegNet [22] achieve high performance on semantic segmentation tasks [23], and lightweight architectures, including MobileNet and EfficientNet, have reduced computational demands [24,25]. However, these approaches still require large labeled datasets and significant computational resources [26,27]. Traditional machine-learning approaches using classifiers such as Support Vector Machines offer practical alternatives, achieving competitive performance with substantially smaller training datasets and lower computational resources [28,29].

In this paper, we present a machine learning-based vegetation segmentation method for desert agriculture using UAV imagery, with the objective of achieving robust segmentation under extreme conditions while maintaining practical deployability with limited training data. Desert environments present distinct challenges, including high spectral similarity between vegetation and soil, extreme illumination variations, and limited color contrast. To address these challenges, we integrate HSV color features [30] with Gray-Level Co-occurrence Matrix (GLCM) texture descriptors [31]. The HSV color space separates chrominance from luminance, providing robustness to illumination variations, while GLCM features enhance discrimination capability by capturing spatial relationships that distinguish vegetation from spectrally similar backgrounds. Support Vector Machine (SVM) with radial basis function kernel [32] is employed for classification due to its effectiveness with limited training samples. The proposed method is evaluated against spectral index-based approaches (ExG, CIVE) and a deep learning baseline to validate its effectiveness. Although the employed techniques are well established, this study contributes a problem-driven integration and systematic evaluation tailored to vegetation segmentation in ultra-high-resolution UAV imagery under desert agricultural conditions.

## 2. Materials and Methods

**Figure 1:**
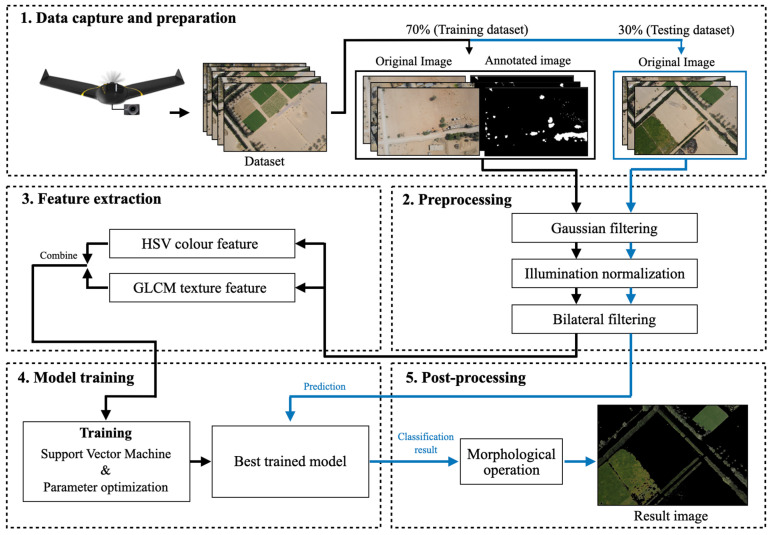
The workflow of the proposed green vegetation segmentation method.

The workflow of the proposed green vegetation segmentation method consists of five main processes: data preparation, preprocessing, feature extraction, model training, and post-processing, as shown in Figure 1.

### 2.1. Data Capture and Preparation

**Figure 2:**
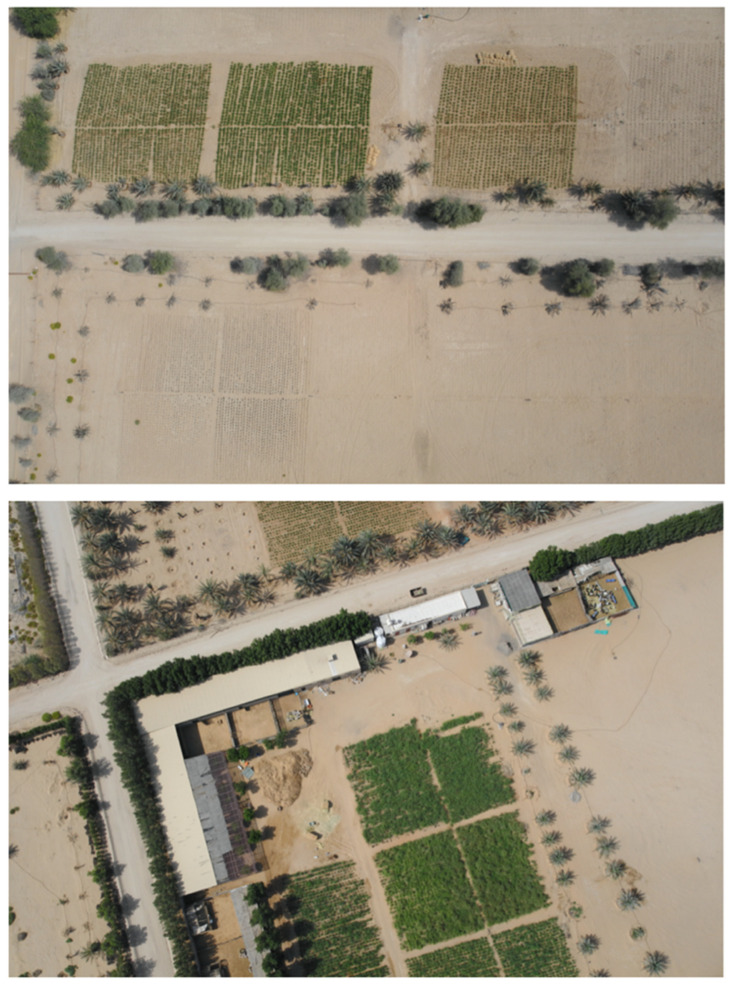
The two examples of the images captured by the drone flying over the farmlands in the Northern Emirates of the UAE.

**Figure 3:**
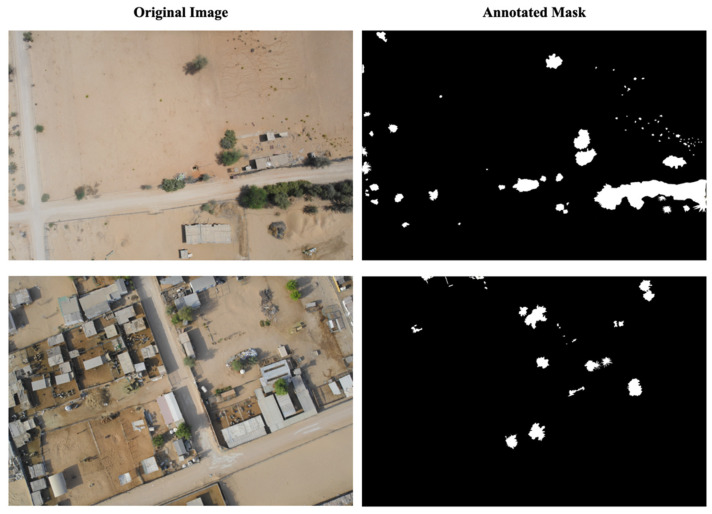
The examples of the original images and their annotated images in the prepared training dataset.

We collected 120 high-resolution images (5472 × 3648 pixels) captured by a senseFly eBee X fixed-wing drone equipped with an onboard senseFly S.O.D.A camera (both from senseFly SA, Cheseaux-sur-Lausanne, Switzerland), flying at a fixed altitude (122 m) over farmlands in the Northern Emirates of the United Arab Emirates, including Sharjah, Ras Al Khaimah, and Ajman. The images were captured between February and March 2023 at different times of day (morning, midday, and afternoon) to ensure diversity in lighting conditions. At this altitude, the ground sampling distance is approximately 2.5 cm/pixel. Figure 2 shows two examples of the images captured by the drone. The dataset was divided into training (70%, 84 images) and testing (30%, 36 images) sets to ensure proper model evaluation and prevent overfitting. To prepare the training data, representative green and non-green regions were manually annotated and selected from vegetation-rich areas within the farmland images using systematic visual interpretation. This selection process naturally resulted in an approximately balanced class distribution (49% green and 51% non-green), as shown in Table 1. Annotations were performed by members of the research team with expertise in agricultural remote sensing and vegetation mapping following systematic visual interpretation. Ambiguous pixels at vegetation boundaries were deliberately excluded to ensure conservative labeling and reduce uncertainty in class assignment. Although this expert-driven annotation process was designed to maximize label quality, no quantitative inter-annotator agreement metric (e.g., Cohen’s kappa) was computed. The absence of a formal quantitative indicator of labeling reliability is therefore acknowledged as a methodological limitation of this study. Figure 3 shows the examples of the original images and their annotated images in the prepared training dataset.

### 2.2. Preprocessing

To improve robustness against varying illumination conditions and sensor noise commonly encountered in drone imagery, we applied several preprocessing steps to enhance image quality before feature extraction. First, Gaussian filtering with σ=1.0 was applied to reduce sensor noise while preserving edge information that was important for accurate segmentation. Second, illumination normalization was performed by applying histogram equalization to the V (value) channel in the HSV color space. This technique redistributes pixel intensities across the full available range by transforming the cumulative distribution function to be approximately uniform, thereby enhancing contrast under varying lighting conditions. The transformation is mathematically defined as follows:

(1) $$V^{\prime }(x,y)=(L-1)\times \mathrm{CDF}(V(x,y))$$

where $V^{\prime }$ is the normalized value channel, L is the number of gray levels (256), and CDF is the cumulative distribution function of the original V channel intensities. Finally, bilateral filtering with parameters $\sigma_{d}=9$ and $\sigma_{r}=75$ were used to reduce noise while maintaining important boundary information between vegetation and background areas. These preprocessing steps ensure that the subsequent feature extraction and classification processes are more robust to illuminance variations, shadows, and sparse vegetation patterns typically found in desert agricultural environments.

The adopted preprocessing pipeline was empirically selected through preliminary experiments to enhance robustness under desert imaging conditions. Gaussian filtering was primarily applied to suppress sensor noise, histogram equalization on the V channel was used to mitigate illumination imbalance, and bilateral filtering was employed to reduce noise while preserving vegetation boundaries. Although a formal sensitivity or ablation analysis of individual preprocessing steps was not conducted, the selected combination represents a practical trade-off between segmentation robustness and computational efficiency for UAV imagery acquired in desert agricultural environments.

### 2.3. Feature Extraction

#### 2.3.1. HSV Color Feature

**Figure 4:**
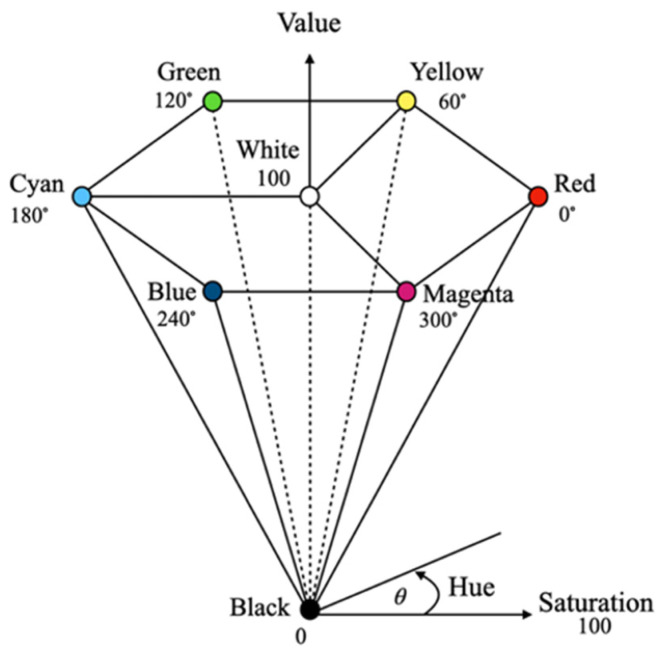
The representation of the HSV color space.

In this study, the HSV color space was used to represent the color features of the images due to its ability to separate chrominance from luminance, providing robustness to the illumination variations commonly found in desert environments. The green and non-green pixel values in the training data were converted from the RGB color space to the HSV color space, which consists of three components: hue (H), saturation (S), and value (V). The HSV color space, proposed by Smith in 1978, enables illumination-invariant feature extraction: hue provides color information that is relatively insensitive to lighting changes, saturation distinguishes vegetation from spectrally similar desaturated backgrounds, and value enables independent brightness normalization. The HSV model can be represented as a hexagonal cone (see Figure 4). The conversion from RGB to HSV follows Equations (2)–(6).

(2) $$m=\max\left(r,g,b\right)$$

(3) $$n=\min\left(r,g,b\right)$$

(4) $$H=\left\{\begin{array}{cc} 0, & if\;m=n\\ \left(60\times \left(\frac{g-b}{m-n}\right)+360\right)\mathrm{mod}\;360, & if\;m=r\\ \left(60\times \left(\frac{b-r}{m-n}\right)+120\right)\mathrm{mod}\;360, & if\;m=g\\ \left(60\times \left(\frac{r-g}{m-n}\right)+240\right)\mathrm{mod}\;360, & if\;m=b \end{array}\right.$$

(5) $$S=\left\{\begin{array}{c} 0,\;\;\;\;\;\;\;\;\;\;\;if\;m=0\\ \frac{m-n}{m},\;\;\mathrm{otherwise} \end{array}\right.$$

(6) V=m

#### 2.3.2. Texture Feature

To enhance discrimination between green vegetation and similarly colored non-vegetation objects (such as green buildings or equipment), we extracted texture features using Gray-Level Co-occurrence Matrix (GLCM) [31]. The GLCM P(i,j) quantifies spatial relationships between pixel intensities by counting the frequency of pixel pairs with gray levels i and j separated by a specified distance d and angle θ, normalized to probability values. We selected d=1 to capture immediate spatial relationships between adjacent pixels, which is suitable for detecting fine-grained texture patterns. This parameter selection was guided by the ultra-high spatial resolution of the UAV image, where fine-scale texture variations are critical for distinguishing vegetation from visually similar background objects.

For each pixel, we computed GLCM features in a 7 × 7 neighborhood window using four orientations (0°, 45°, 90°, 135°), which provides a balance between capturing local texture characteristics and maintaining spatial stability without excessive smoothing. The following texture features, including contrast, homogeneity, energy, and correlation, are extracted from the GLCM to characterize the local texture properties of each pixel. Contrast measures local intensity variation. Homogeneity measures local uniformity. Energy measures textural uniformity. Correlation measures linear dependency of gray levels. These features are calculated following Equations (7)–(10).

(7) $$Contrast=\sum_{i,j}{\left|i-j\right|}^{2}\cdot P\left(i,j\right)$$

(8) $$Homogeneity=\sum_{i,j}\frac{P\left(i,j\right)}{1+\left|i-j\right|}$$

(9) $$Energy=\sum_{i,j}P{\left(i,j\right)}^{2}$$

(10) $$Correlation=\sum_{i,j}\frac{\left(i-\mu_{i}\right)\left(j-\mu_{j}\right)P\left(i,j\right)}{\sigma_{i}\sigma_{j}}$$

where $P\left(i,j\right)$ is the GLCM, μ and σ are the mean and standard deviation of the marginal distributions. The final feature vector for each pixel combines color and texture information as follows:

(11) $$f={\left[H,S,V,Contrast,Homogeneity,Energy,Correlation\right]}^{T}$$

### 2.4. Model Training

#### 2.4.1. Training Phase

**Figure 5:**
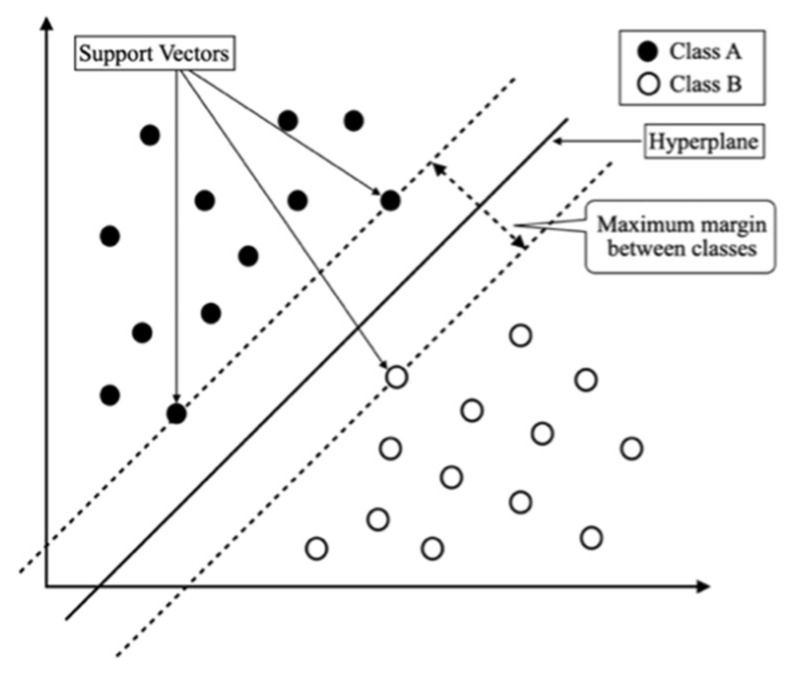
An example of SVM dealing with a two-class classification in a two-dimensional space.

In the training phase, the support vector machine (SVM) classifier was used as a learning algorithm to learn the HSV color features and texture features of the green and non-green color pixels in the training data. SVM creates an optimal hyperplane to separate green and non-green classes by maximizing the margin between support vectors [32]. Figure 5 shows an example of an SVM performing a two-class classification in two-dimensional space.

For our non-linearly separable data, we employed the Radial Basis Function (RBF) kernel to find the optimal hyperplane, which has shown superior performance for remote sensing applications [33]:

(12) $$K\left(x_{i},x_{j}\right)=exp\left(-\gamma {\left||x_{i}-x_{j}|\right|}^{2}\right)$$

The optimization problem with a soft margin to handle noise and outliers is calculated as follows:

(13) $$\underset{w,b,\xi }{\min}\frac{1}{2}{\left||w|\right|}^{2}+C\sum_{i=1}^{N}\xi_{i}$$

Subject to the following:

(14) $$y_{i}\left(w\cdot \phi \left(x_{i}\right)+b\right)\geq 1-\xi_{i},\;\xi_{i}\geq 0,\;i=1,\ldots ,N$$

where w is the weight vector; b is the bias; $\xi_{i}$ are slack variables; C is the regularization parameter; and ϕ is the kernel mapping function.

#### 2.4.2. Parameter Optimization

SVM hyperparameters (C and γ) were optimized using 5-fold cross-validation on the training set with grid search to prevent overfitting. The regularization parameter C was tested over the range $C\in \left\{0.1,1,10,100,1000\right\}$, while the RBF kernel parameter γ was evaluated over the range $\gamma \in \left\{0.001,0.01,0.1,1,10\right\}$. The parameter combination yielding the highest cross-validation F1-score was selected to ensure optimal performance on unseen data. Through this systematic optimization process, the optimal parameters were determined to be C=100 and γ=0.1.

To mitigate spatial autocorrelation effects, training and testing samples were derived from spatially independent UAV images, and no pixels from the same image were shared between the training and testing sets. The 5-fold cross-validation was applied exclusively within the training data for hyperparameter tuning. Nevertheless, as the classification is performed at the pixel level, residual spatial dependency within individual images cannot be entirely eliminated and is therefore acknowledged as a methodological limitation of this study.

### 2.5. Post-Processing

To improve segmentation quality and remove noise artifacts, we applied morphological operations to refine the classification results. First, an opening operation was performed to remove small noise artifacts using a 3 × 3 circular structural element, which helped eliminate isolated pixels that were incorrectly classified as vegetation. Second, a closing operation was applied to fill small gaps within vegetation areas using a 5 × 5 circular structural element, ensuring that fragmented vegetation regions are properly connected. Finally, a 3 × 3 median filter was applied to smooth boundaries while preserving the overall shape of vegetation areas. These post-processing steps enhanced the final segmentation quality by reducing false positives and creating more coherent vegetation regions that better represent the actual green areas in the drone imagery.

## 3. Results and Discussion

### 3.1. Evaluation Metrics and Baseline Methods

We evaluated performance using comprehensive metrics that provided different perspectives on segmentation quality. Accuracy measured overall pixel classification correctness, precision represented the fraction of predicted green pixels that were actually green, recall indicates the fraction of actual green pixels correctly identified, F1-score provided the harmonic mean of precision and recall, and Intersection over Union (IoU) measured the overlap between predicted and ground truth regions. The mathematical formulations were defined as follows:

(15) $$Accuracy=\frac{TP+TN}{TP+TN+FP+FN}$$

(16) $$Precision=\frac{TP}{TP+FP}$$

(17) $$Recall=\frac{TP}{TP+FN}\;$$

(18) $$F1=\frac{2\times Precision\times Recall}{Precision+Recall}$$

(19) $$IoU=\frac{TP}{TP+FP+FN}$$

where TP, TN, FP, FN represent True Positives, True Negatives, False Positives, and False Negatives, respectively.

The proposed method was compared against five baseline approaches to ensure comprehensive evaluation. The ExG method calculated ExG=2G−R−B followed by Otsu thresholding. The CIVE method computed CIVE=0.441R−0.811G+0.385B+18.78745 followed by Otsu thresholding. The U-Net baseline used a pre-trained model fine-tuned on our dataset, representing a state-of-the-art deep learning approach. The HSV-only SVM method employed our SVM approach using only HSV features for an ablation study, while the RGB SVM trained SVM on raw RGB features for a comparison study.

All experiments were conducted using MATLAB R2021b on a workstation with Intel Core i7-10700K CPU, 32 GB RAM, and NVIDIA RTX 3080 GPU. For the U-Net baseline, we used a pre-trained model from the Computer Vision Toolbox and fine-tuned it using transfer learning with specific parameters optimized for our agricultural segmentation task. The learning rate was set to $1\times 10^{-4}$ with a batch size of 8 images to balance training stability and memory constraints. Training was performed for 50 epochs using the Adam optimizer, which provided good convergence characteristics for our dataset. To improve generalization and prevent overfitting, data augmentation techniques, including random rotation, scaling, and horizontal flipping, were applied during the training process. All methods, including spectral index-based approaches, SVM-based models, and the U-Net baseline, were evaluated using the same training–testing split and identical test images to ensure a fair and consistent comparison.

### 3.2. Visual Performance Comparison

**Figure 6:**
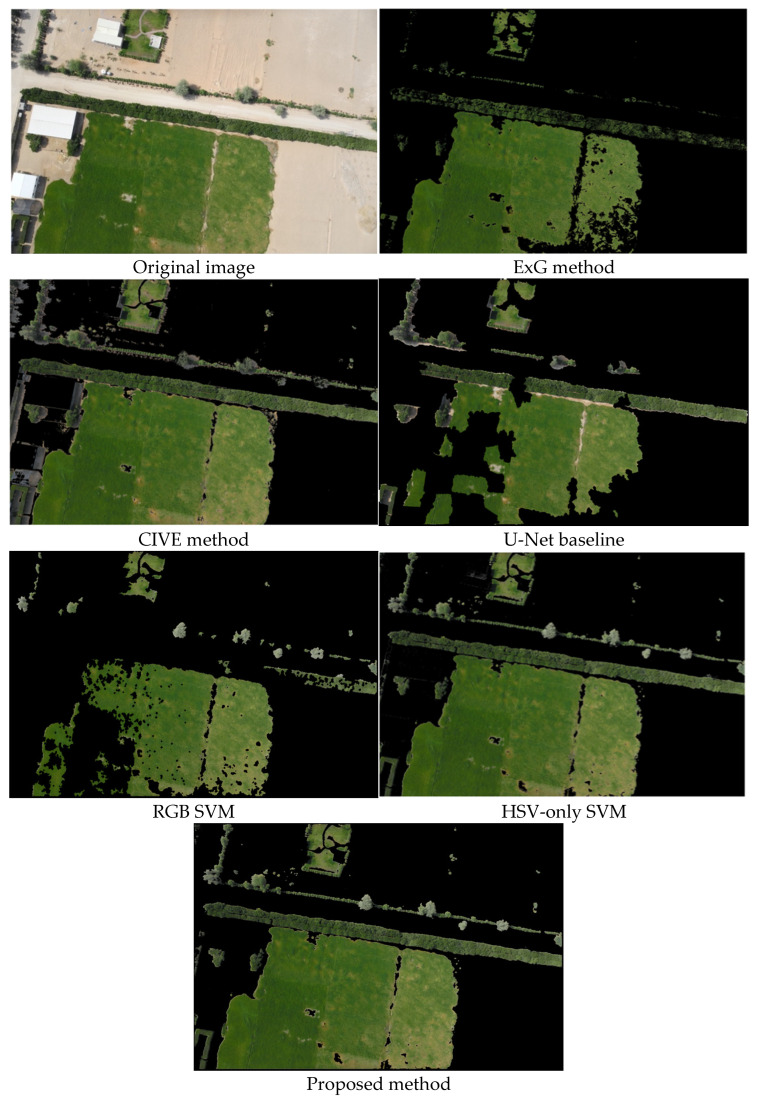
The results of the green area segmentation in drone imagery of the six different methods.

**Figure 7:**
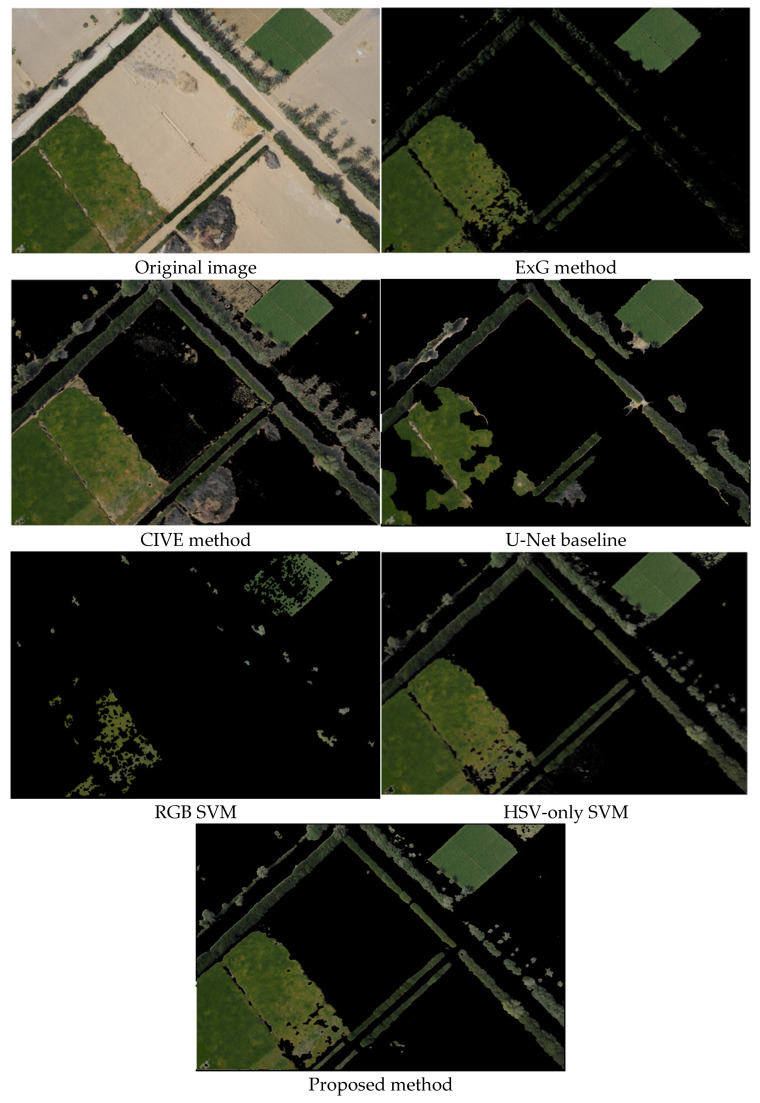
The results of the green area segmentation in drone imagery of the six different methods.

**Figure 8:**
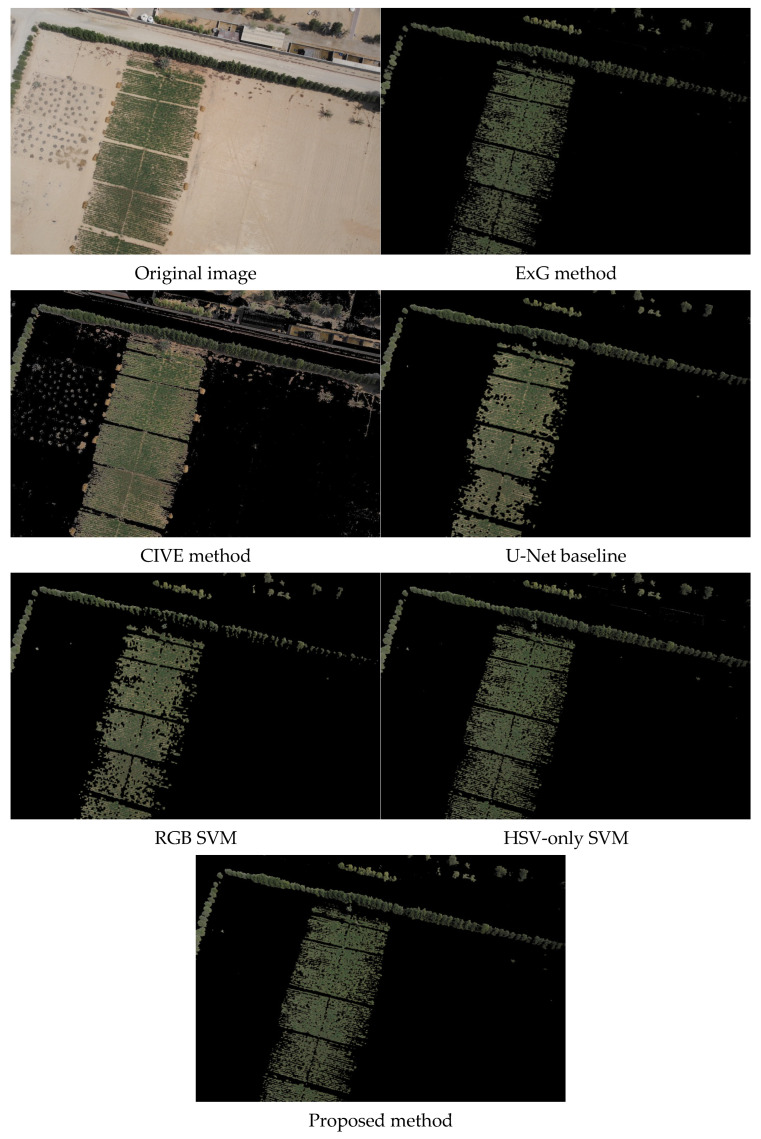
The results of the green area segmentation in drone imagery of the six different methods.

To illustrate method behavior, we applied all six methods to three representative test images. Figure 6, Figure 7 and Figure 8 demonstrate different scenarios encountered in desert agriculture: dense vegetation with building shadows and infrastructure (Figure 6), sparse scattered vegetation with high soil-vegetation contrast (Figure 7), and organized row crop patterns with complex boundaries (Figure 8). These scenarios were selected to highlight performance differences across varying conditions. Comprehensive quantitative evaluation with objective metrics (precision, recall, F1-score, IoU) across all 36 test images is provided in Section 3.3.

The visual analysis across test images reveals distinct performance patterns among the methods. Traditional spectral index methods (ExG and CIVE) struggled significantly with challenging conditions, producing noise, false positives in soil regions, and failing to detect vegetation in shadowed areas, with ExG showing particularly limited effectiveness for sparse, scattered vegetation (Figure 7). The RGB SVM method demonstrated fragmented results with difficulty maintaining spatial coherence, while the U-Net baseline performed well overall but occasionally over-segmented non-vegetation areas (Figure 6) and missed some smaller scattered patches. The HSV-only SVM method improved upon RGB-based approaches with cleaner boundaries, demonstrating the effectiveness of HSV color space transformation. The proposed method achieved the most accurate segmentation across all three scenarios, identifying green vegetation in shadowed areas where traditional methods failed, successfully detecting small scattered green patches typical in desert agriculture, maintaining clear boundary delineation with reduced noise artifacts in organized crop rows (Figure 8).

### 3.3. Quantitative Performance Comparison

The quantitative evaluation was conducted on 36 test images with manually generated ground truth annotations. Ground truth segmentation was performed by experienced agricultural remote sensing researchers following systematic visual interpretation protocols. Each pixel was assigned a binary label (1 = green vegetation, 0 = non-vegetation) based on characteristic green coloration and its spatial context within cultivated areas. Ambiguous pixels along vegetation boundaries were carefully reviewed and labeled conservatively to ensure ground truth reliability. The segmentation results of all six methods were then compared against the ground truth labels using comprehensive evaluation metrics.

Table 2 summarizes the quantitative results of all six methods. The results show that the proposed method achieves the highest performance across all evaluation metrics. The RGB SVM method shows the lowest performance with an accuracy of 0.75 and an IoU of 0.59, reflecting the limitations of the RGB color space for desert green vegetation segmentation. ExG method performs slightly better with an accuracy of 0.78 and an IoU of 0.61 but still exhibits excessive noise and false positives visible in the segmentation images. CIVE method achieves moderate performance with an accuracy of 0.81 and an IoU of 0.65, consistent with its cleaner but still limited segmentation capability. The U-Net baseline demonstrates good performance (accuracy 0.88, IoU 0.74), matching its strong visual results, though still below the proposed method. HSV-only SVM shows substantial improvement (accuracy 0.89, IoU 0.75) over RGB-based methods, confirming the effectiveness of HSV color space transformation. The proposed method achieves the highest scores (accuracy 0.91, F1-score 0.88, IoU 0.82), representing improvements of 14% in F1-score and 21% in IoU over ExG, and 11% in F1-score and 17% in IoU over CIVE, demonstrating the value of incorporating GLCM texture features alongside HSV color information.

The computational efficiency analysis revealed that the proposed method provides a practical trade-off between accuracy and computational requirements. While traditional spectral index methods are fastest (0.12–0.15 s per image), they provided significantly lower accuracy. The proposed method required 25 s per image processing time compared to 2.1 s for the U-Net baseline but achieved superior segmentation accuracy with much lower memory requirements (130 MB compared to 2.8 GB for the deep learning approach). The training time of 28 min was substantially lower than the 45 min required for the U-Net baseline, making the proposed method more accessible for deployment in resource-constrained environments.

These results indicate that the proposed method prioritizes segmentation accuracy over processing speed, while offering competitive performance compared to modern deep learning approaches with substantially lower computational requirements during training and deployment. The combination of high accuracy, reduced memory demand, and practical deployability suggests potential applicability in precision agriculture applications in arid regions, where robust vegetation detection is prioritized over real-time processing, such as irrigation management, crop monitoring, and yield estimation.

## 4. Conclusions

In this paper, we present a robust machine learning approach for identifying green vegetation areas in desert UAV imagery by integrating HSV color features with GLCM texture descriptors using Support Vector Machine classification. Experimental results indicate improved performance compared to traditional spectral index-based methods and competitive results relative to modern deep learning approaches, achieving an accuracy of 0.91, an F1-score of 0.88, and an IoU of 0.82 with statistical significance (*p* < 0.05 for all comparisons), while requiring substantially lower computational resources. The combination of high segmentation accuracy, moderate processing time, and robustness to challenging desert conditions, such as shadows, variable soil appearance, and sparse vegetation, suggests potential applicability in precision agriculture tasks in arid environments. In particular, accurate vegetation delineation provided by the proposed method shows strong potential for supporting UAV-based precision spraying applications, where precise targeting is essential for optimizing spray deposition and reducing off-target losses, as highlighted in recent studies on UAV-assisted precision spraying systems [34]. Future work will focus on incorporating multi-spectral information and improving computational efficiency to enable near real-time deployment in autonomous agricultural systems.

## Figures and Tables

**Table 1:** The number of green and non-green selected pixels in the training dataset.

Color	Number of Pixels	Percentage
Green	132,844	49%
Non-green	138,268	51%
Total	271,112	100%

**Table 2:** Comprehensive performance comparison on the test dataset.

Method	Accuracy	Precision	Recall	F1-Score	IoU
ExG	0.78	0.72	0.76	0.74	0.61
CIVE	0.81	0.75	0.79	0.77	0.65
U-Net baseline	0.88	0.85	0.84	0.85	0.74
RGB SVM	0.75	0.71	0.73	0.72	0.59
HSV-only SVM	0.89	0.86	0.85	0.86	0.75
Proposed method	0.91	0.89	0.87	0.88	0.82

## Data Availability

The original contributions presented in this study are included in the article. Further inquiries can be directed to the corresponding author.
